# Crystal structure of diethyl 3,3′-{2,2′-(1*E*)-[1,4-phenyl­enebis(azan-1-yl-1-yl­idene)]bis­(methan-1-yl-1-yl­idene)bis­(1*H*-pyrrole-2,1-di­yl)}di­propano­ate

**DOI:** 10.1107/S2056989015005113

**Published:** 2015-03-25

**Authors:** Jasim Alshawi, Muoayed Yousif, Gregore Timco, Inigo J. Vitorica Yrezabal, Richard Winpenny, Mohamad J. Al-Jeboori

**Affiliations:** a Department of Chemistry, College of Education for Pure Science, University of Basrah, Iraq; b School of chemistry, University of Manchester, Oxford Road, Manchester, M13 9PL, UK; c Department of Chemistry, College of Education (Ibn Al-Haitham) for Pure Science, University of Baghdad, Iraq

**Keywords:** crystal structure, Schiff base, bis­(pyrrole ester)

## Abstract

The complete mol­ecule of the title compound, C_26_H_30_N_4_O_4_, is generated by crystallographic inversion symmetry. The dihedral angle between the planes of the benzene and pyrrole rings is 45.20 (11)°; the N atom bonded to the the benzene ring and the pyrrole N atom are in a *syn* conformation. The side chain adopts an extended conformation [N—C—C—C = 169.07 (17)° and C—O—C—C = −176.54 (17)°]. No directional inter­actions could be identified in the crystal packing.

## Related literature   

For the synthesis of di­pyrrole Schiff base ligands, see: Meghdadi *et al.*(2010 ▸); Munro *et al.* (2004 ▸). For the synthesis of pyrrole ester precursors, see: Koriatopoulou *et al.* (2008 ▸); Singh & Pal (2010 ▸). For the preparation of the title compound, see: Yang *et al.* (2004 ▸); Ourari *et al.* (2013 ▸).
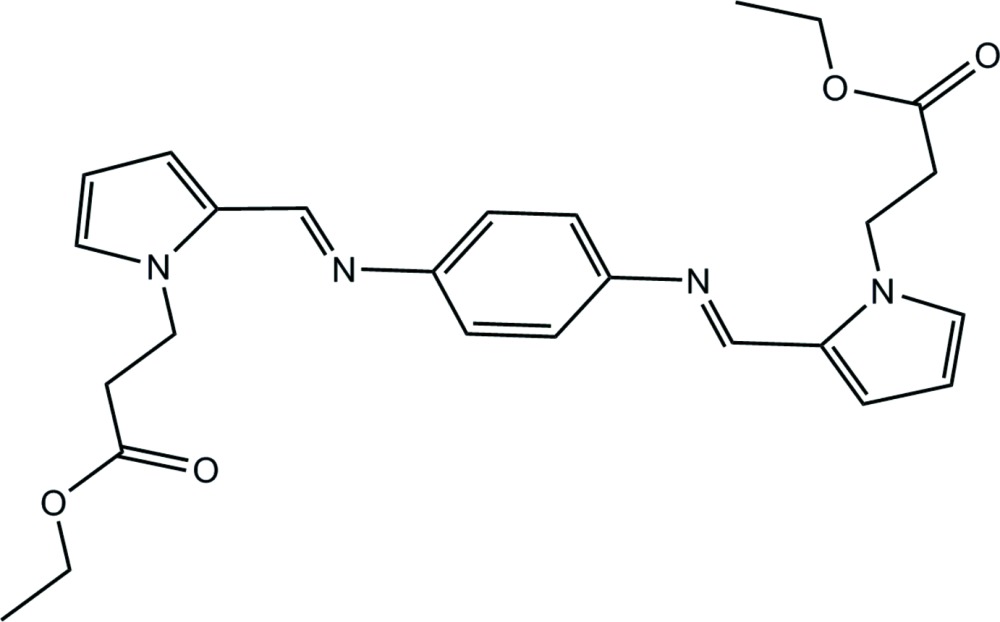


## Experimental   

### Crystal data  

C_26_H_30_N_4_O_4_
*M* *_r_* = 462.54
Monoclinic, 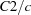

*a* = 21.6153 (10) Å
*b* = 8.1227 (4) Å
*c* = 13.9404 (8) Å
β = 94.395 (5)°
*V* = 2440.4 (2) Å^3^
*Z* = 4
Mo *K*α radiation
μ = 0.09 mm^−1^
*T* = 150 K
0.4 × 0.3 × 0.3 mm

### Data collection  

Agilent SuperNova (Single source at offset, Atlas) diffractometer
Absorption correction: multi-scan (*CrysAlis PRO*; Agilent, 2013 ▸) *T*_min_ = 0.613, *T*_max_ = 1.000
6592 measured reflections
2900 independent reflections
1697 reflections with *I* > 2σ(*I*)
*R*_int_ = 0.063

### Refinement  

*R*[*F* ^2^ > 2σ(*F* ^2^)] = 0.055
*wR*(*F* ^2^) = 0.138
*S* = 1.10
2900 reflections
155 parameters
H-atom parameters constrained
Δρ_max_ = 0.22 e Å^−3^
Δρ_min_ = −0.26 e Å^−3^

### 

Data collection: *CrysAlis PRO* (Agilent, 2013 ▸); cell refinement: *CrysAlis PRO*; data reduction: *CrysAlis PRO*; program(s) used to solve structure: *SHELXS97* (Sheldrick, 2008 ▸); program(s) used to refine structure: *SHELXL2014* (Sheldrick, 2015 ▸); molecular graphics: *OLEX2* (Dolomanov *et al.*, 2009 ▸); software used to prepare material for publication: *OLEX2*.

## Supplementary Material

Crystal structure: contains datablock(s) I. DOI: 10.1107/S2056989015005113/hb7371sup1.cif

Structure factors: contains datablock(s) I. DOI: 10.1107/S2056989015005113/hb7371Isup2.hkl

**Fig. 1.**
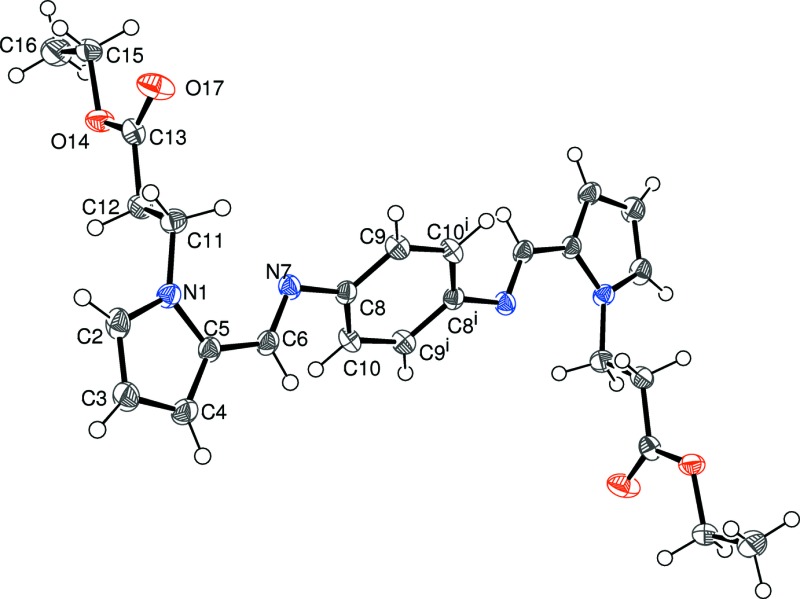
A view of the molecular structure of the title compound. Displacement ellipsoids are drawn at the 50% probability level.

. DOI: 10.1107/S2056989015005113/hb7371fig1.tif
A view of the mol­ecular structure of the title compound. Displacement ellipsoids are drawn at the 50% probability level.

CCDC reference: 1053761

Additional supporting information:  crystallographic information; 3D view; checkCIF report

## supplementary crystallographic information

### S1. Comment

The Schiff base diethyl 3,3'-(2,2'-(1E)-(1,4-phenylenebis(azan-1-yl-1-ylidene))bis (methan-1-yl-1-ylidene)bis(1H-pyrrole-2,1-diyl))dipropanoate was prepared in two steps. The reaction of 1H-pyrrole-2-carbaldehyde with ethyl bromopropanoate resulted in the formation of (2-formyl-1H-pyrrole-1-yl)-propanoate (Koriatopoulou *et al.* (2008) and Singh & Pal (2010)). The reaction of two moles of the pyrrole ester with p-phenylenediamine gave the title of Schiff-base compound (Yang *et al.*, 2004; Ourari *et al.*, 2013). The compound with molar mass 462.54 g mol-1, crystallizes in monoclinic crystal structure with a space group c12 /c1 and had a calculated density 1.259 g cm-3. The asymetric unit consists of half the molecule, the molecule is completed by inversion symmetry. Infrared spectra indidicates typical absorbance bands of the

functional -C=N and carbonyl -C=O at 1595 and 1680 cm-1, respectively. The positive ES mass spectrum of the bis Schiff base showed a parent ion peak at m/z = 463.7 (M+H)+, corresponding to C26H30N4O4, for which the required value=462.3. The values distance (1.270Å) observed for (N7-C6) is shorter than (N7-C8) (1.458Å), indicating a double bond order. The distance observed at (1.384Å) for (N1-C5), revealed a resonance is occurred in the pyrrole system between lon pair electron of the nitrogen atom and the pyrrole ring. Other bond lengths and bond angles are within reported values.

### S2. Experimental

FT-IR data were recorded on Agilent 8400s FT-IR while NMR data were collected on Bruker 500 MHz spectrometer in CDCl3 solutions. The assignment of the chemical shifts for the NMR data were made following numbering shown in structure. The title compound was prepared in two steps and as follows: Preparation of ethyl(2-formyl-1*H*-pyrrole-1-yl)-propanoate(L): It was prepared by literature procedures (Koriatopoulou *et al.*, (2008); Singh & Pal (2010), as follows: To a mixture of 1H-pyrrole-2-carbaldehyde(1.00g,10.51mmol), K_2_CO_3_ (2.90g, 21.02mmol) and (2.64g, 10.51mmol) of 18-crown-6 in dry 1,4-dioxane (20ml), was added a solution of ethyl bromopropanoate (2.17g, 12mmol) in dry 1,4-dioxane (20ml)dropwise over a period of 30 min.The reaction mixture was allowed to reflux under nitrogen atmosphere for 6h, and then the solvent was removed under reduced pressure. Water (50ml) was added to the residue, and the mixture was extracted with ethyl acetate (3 × 15ml). The combined organic layers were washed with brine (15ml), and then dried over Na_2_SO_4_.The solvent was removed under reduced pressure, and the oily residue was purified by flash chromatography with an eluent mixture (33% ethyl acetate / hexane), yield 0.70 g (70%) of the title compound as a yellow oil product.IR (ATR cm^-1^): 1660 ν(C=O) aldehyde moiety. 172 0 ν (C=O) ester group. NMR data (p.p.m),δH (500 MHz, CDCl_3_): 1.10 (3H, t, C13-H), 2.70 (2H, t, C9-H), 4.01 (2H, Q, C12-H), 4.47 (2H, t, C8-H), 6.09 (1H, t, C3-H),6.83 (1H, d, C4-H), 6.94 (1H, d, C2-H) and 9.43 (1H, s, C6-H); δC (125.75 MHz, CDCl3):14.06 C13, 35.68 C9, 44.71 C8, 60.62 C12,109.53 C3, 125.17 C 4, 131.02 C5 and 132.23 C2, and C=O of the carboxylate moiety at 171.17 (C10) and 179.15 for C6. The positive ES mass spectrum at m/z =196.4 (M+H)^+^ (80 %) for C10H13NO3, requires =195.1. The other peaks which detected at m/z =167.4 (100 %), 123.3 (50 %), 95.2 (5 %) and 67 (6 %) correspond to [M-CH2CH3]^+^, [M-(CH2CH3+CO2)]^+^, [M-(CH2CH3+CO2+CH2+CH2)]^+^ and [M-(CH2CH3+CO2+CH2CH2+CO)]^+^, respectively.Synthesis of the title Schiff-base:achieved using standard method as follows: To a mixture of L (1.95g, 10mmol) in ethanol (20ml)with 3 drops of glacial acetic acid, a solution of p-phenylendiamine (0.5g, 5mmol) in ethanol (20ml) was added dropwise over a period of 20 min. The reaction mixture was allowed to reflux for 3h, and then cooled to room temperature. A white precipitate was collected by filtration and recrystallised from ethanol, yield 1.07g (55%). Crystals were obtained from evaporation of a mixture of methanol/acetone at room temperature. IR (ATR,cm^-1^): at line % / 1595 (C=N),1680 (C=O). ^1^H NMR dH (500 MHz, CDCl_3_, p.p.m) δH: 1.16 (6H, t, C16, 16–H), 2.85 (4H, t, C12, 12–H), 4.05 (4H, q, C15, 15–H), 4.67 (4H, t, C11,11–H), 6.11 (2H, t, C3, 3–H), 6.58 (2H, d, C4,4–H), 6.83 (2H, d, C2, 2–H), 7.11 (4H, s, C9, 9, C10, 10–H) and 8.25 (2H, s, C6, 6–H): ^13^C NMR (125.75 MHz, CDCl_3_, p.p.m) δC: 14.29 (C16, 16-), 36.14 (C12, 12-), 44.98 (C11, 11-), 60.62 (C15,15-), 108.85 (C 3, 3-), 119.88 (C2, 2-), 121.57 (C9,8-) and C10,10-), 129.16 and 129.17 to (C4, 4- and C5, 5-), 149.38 (C6, 6-) and 150.02 (C 8, 8-). C=O at 171.12 (C13,13-). ). The positive ES mass spectrum at m/z = 463.7 (M+H)^+^ (42%) for C26H30N4O4, requires =462.3. The other peaks detected at m/z =405.2 (3%), 361.6 (12%), 317.2 (3%) and 261.4 (3%) correspond to [M-2(CH2CH3)]^+^, [M-(2(CH2CH3)+CO2)]^+^, [M-(2CH2CH3+2CO2)]^+^ and [M-(2CH2CH3+2CO2+2CH2CH2)]^+^, espectively.

### S3. Refinement

Crystal data, data collection and structure refinement details are summarized in Table 1.

The refinement was H atom attached to N1 was located in the difference Fourier map and refined isotropically. All other H atoms were placed in calculated positions with d(C—H) = 0.95 Å for aromatic, 0.99 for CH2 and 0.98 Å for CH3 atoms. The Uiso values were constrained to be 1.5Ueq of the carrier atom for methyl H atoms and 1.2Ueq for the remaining H atoms. A rotating group model was used for the methyl groups.

### Crystal data

**Table:**

C_26_H_30_N_4_O_4_	*F*(000) = 984
*M**_r_* = 462.54	*D*_x_ = 1.259 Mg m^−^^3^
Monoclinic, *C*2/*c*	Mo *K*α radiation, λ = 0.71073 Å
*a* = 21.6153 (10) Å	Cell parameters from 1750 reflections
*b* = 8.1227 (4) Å	θ = 3.8–27.5°
*c* = 13.9404 (8) Å	µ = 0.09 mm^−^^1^
β = 94.395 (5)°	*T* = 150 K
*V* = 2440.4 (2) Å^3^	Block, clear light colourless
*Z* = 4	0.4 × 0.3 × 0.3 mm

### Data collection

**Table:**

Agilent SuperNova (Single source at offset, Atlas) diffractometer	2900 independent reflections
Radiation source: SuperNova (Mo) X-ray Source	1697 reflections with *I* > 2σ(*I*)
Mirror monochromator	*R*_int_ = 0.063
Detector resolution: 10.3705 pixels mm^-1^	θ_max_ = 29.5°, θ_min_ = 2.9°
ω scans	*h* = −18→29
Absorption correction: multi-scan (*CrysAlis PRO*; Agilent, 2013)	*k* = −10→7
*T*_min_ = 0.613, *T*_max_ = 1.000	*l* = −19→18
6592 measured reflections	

### Refinement

**Table:**

Refinement on *F*^2^	Primary atom site location: structure-invariant direct methods
Least-squares matrix: full	Hydrogen site location: inferred from neighbouring sites
*R*[*F*^2^ > 2σ(*F*^2^)] = 0.055	H-atom parameters constrained
*wR*(*F*^2^) = 0.138	*w* = 1/[σ^2^(*F*_o_^2^) + (0.0338*P*)^2^ + 0.0686*P*] where *P* = (*F*_o_^2^ + 2*F*_c_^2^)/3
*S* = 1.10	(Δ/σ)_max_ < 0.001
2900 reflections	Δρ_max_ = 0.22 e Å^−^^3^
155 parameters	Δρ_min_ = −0.26 e Å^−^^3^
0 restraints	

### Special details

**Table:**

Experimental. CrysAlisPro, Agilent Technologies, Version 1.171.36.28 (release 01-02-2013 CrysAlis171 .NET) (compiled Feb 1 2013,16:14:44) Empirical absorption correction using spherical harmonics, implemented in SCALE3 ABSPACK scaling algorithm.
Geometry. All e.s.d.'s (except the e.s.d. in the dihedral angle between two l.s. planes) are estimated using the full covariance matrix. The cell e.s.d.'s are taken into account individually in the estimation of e.s.d.'s in distances, angles and torsion angles; correlations between e.s.d.'s in cell parameters are only used when they are defined by crystal symmetry. An approximate (isotropic) treatment of cell e.s.d.'s is used for estimating e.s.d.'s involving l.s. planes.

### Fractional atomic coordinates and isotropic or equivalent isotropic displacement parameters (Å^2^)

**Table:**

	*x*	*y*	*z*	*U*_iso_*/*U*_eq_	
O14	0.34059 (6)	0.02364 (17)	0.24674 (10)	0.0286 (4)	
N1	0.45878 (7)	0.29578 (19)	0.01458 (12)	0.0225 (4)	
O17	0.41066 (7)	0.2126 (2)	0.29870 (11)	0.0385 (4)	
N7	0.34834 (7)	0.5212 (2)	0.01854 (13)	0.0231 (4)	
C13	0.38269 (9)	0.1407 (2)	0.23355 (16)	0.0240 (5)	
C6	0.37626 (9)	0.4878 (2)	−0.05699 (16)	0.0233 (5)	
H6	0.3615	0.5387	−0.1140	0.028*	
C5	0.42829 (9)	0.3787 (2)	−0.06151 (16)	0.0227 (5)	
C8	0.29823 (9)	0.6351 (2)	0.00733 (15)	0.0213 (5)	
C9	0.29548 (9)	0.7593 (2)	0.07499 (16)	0.0242 (5)	
H9	0.3258	0.7654	0.1260	0.029*	
C2	0.50726 (9)	0.2115 (3)	−0.01898 (17)	0.0285 (5)	
H2	0.5353	0.1466	0.0181	0.034*	
C11	0.44300 (9)	0.2904 (3)	0.11490 (15)	0.0248 (5)	
H11A	0.4308	0.3996	0.1345	0.030*	
H11B	0.4794	0.2581	0.1556	0.030*	
C12	0.39047 (9)	0.1701 (3)	0.12900 (15)	0.0247 (5)	
H12A	0.3521	0.2135	0.0983	0.030*	
H12B	0.3991	0.0663	0.0983	0.030*	
C15	0.33162 (10)	−0.0179 (3)	0.34693 (16)	0.0307 (6)	
H15A	0.3205	0.0796	0.3820	0.037*	
H15B	0.3694	−0.0636	0.3782	0.037*	
C10	0.25193 (9)	0.6253 (2)	−0.06747 (16)	0.0244 (5)	
H10	0.2528	0.5411	−0.1126	0.029*	
C4	0.45880 (9)	0.3440 (3)	−0.14264 (17)	0.0283 (5)	
H4	0.4484	0.3843	−0.2042	0.034*	
C3	0.50810 (10)	0.2377 (3)	−0.11602 (17)	0.0318 (6)	
H3	0.5361	0.1933	−0.1564	0.038*	
C16	0.28000 (11)	−0.1429 (3)	0.34458 (18)	0.0412 (6)	
H16A	0.2913	−0.2375	0.3085	0.062*	
H16B	0.2427	−0.0953	0.3147	0.062*	
H16C	0.2731	−0.1756	0.4091	0.062*	

### Atomic displacement parameters (Å^2^)

**Table:**

	*U*^11^	*U*^22^	*U*^33^	*U*^12^	*U*^13^	*U*^23^
O14	0.0361 (8)	0.0298 (9)	0.0205 (9)	−0.0087 (7)	0.0052 (7)	−0.0004 (7)
N1	0.0217 (9)	0.0226 (10)	0.0236 (10)	0.0014 (7)	0.0051 (8)	0.0034 (8)
O17	0.0397 (9)	0.0498 (11)	0.0266 (10)	−0.0165 (8)	0.0054 (8)	−0.0087 (8)
N7	0.0234 (9)	0.0209 (9)	0.0255 (10)	0.0023 (7)	0.0057 (8)	−0.0010 (8)
C13	0.0213 (11)	0.0228 (11)	0.0283 (13)	−0.0002 (9)	0.0032 (10)	−0.0042 (10)
C6	0.0258 (11)	0.0184 (11)	0.0260 (12)	−0.0029 (8)	0.0042 (10)	0.0040 (10)
C5	0.0241 (10)	0.0189 (11)	0.0257 (12)	0.0002 (8)	0.0064 (9)	0.0021 (10)
C8	0.0212 (10)	0.0192 (11)	0.0242 (12)	0.0008 (8)	0.0071 (9)	0.0028 (10)
C9	0.0209 (10)	0.0260 (12)	0.0255 (12)	−0.0006 (9)	0.0005 (9)	−0.0031 (10)
C2	0.0236 (11)	0.0255 (12)	0.0375 (14)	0.0074 (9)	0.0106 (10)	0.0027 (11)
C11	0.0236 (10)	0.0270 (12)	0.0239 (12)	0.0000 (9)	0.0019 (9)	0.0010 (10)
C12	0.0262 (11)	0.0254 (12)	0.0226 (12)	0.0010 (9)	0.0023 (9)	−0.0028 (10)
C15	0.0414 (13)	0.0290 (13)	0.0230 (13)	−0.0002 (10)	0.0102 (11)	0.0002 (11)
C10	0.0260 (11)	0.0219 (11)	0.0257 (13)	0.0001 (9)	0.0040 (10)	−0.0070 (10)
C4	0.0323 (12)	0.0261 (12)	0.0275 (13)	0.0012 (9)	0.0099 (10)	0.0063 (11)
C3	0.0330 (12)	0.0293 (13)	0.0351 (15)	0.0075 (10)	0.0153 (11)	0.0018 (11)
C16	0.0551 (16)	0.0357 (14)	0.0347 (15)	−0.0100 (11)	0.0152 (13)	0.0024 (12)

### Geometric parameters (Å, º)

**Table:**

O14—C13	1.338 (2)	C2—C3	1.371 (3)
O14—C15	1.464 (2)	C11—H11A	0.9700
N1—C5	1.381 (3)	C11—H11B	0.9700
N1—C2	1.364 (2)	C11—C12	1.522 (3)
N1—C11	1.465 (3)	C12—H12A	0.9700
O17—C13	1.203 (2)	C12—H12B	0.9700
N7—C6	1.282 (2)	C15—H15A	0.9700
N7—C8	1.424 (2)	C15—H15B	0.9700
C13—C12	1.499 (3)	C15—C16	1.507 (3)
C6—H6	0.9300	C10—C9^i^	1.387 (3)
C6—C5	1.437 (3)	C10—H10	0.9300
C5—C4	1.381 (3)	C4—H4	0.9300
C8—C9	1.386 (3)	C4—C3	1.399 (3)
C8—C10	1.391 (3)	C3—H3	0.9300
C9—H9	0.9300	C16—H16A	0.9600
C9—C10^i^	1.387 (3)	C16—H16B	0.9600
C2—H2	0.9300	C16—H16C	0.9600
			
C13—O14—C15	115.83 (16)	C12—C11—H11B	109.2
C5—N1—C11	127.98 (16)	C13—C12—C11	111.61 (17)
C2—N1—C5	108.38 (18)	C13—C12—H12A	109.3
C2—N1—C11	123.62 (18)	C13—C12—H12B	109.3
C6—N7—C8	116.70 (18)	C11—C12—H12A	109.3
O14—C13—C12	112.02 (18)	C11—C12—H12B	109.3
O17—C13—O14	123.3 (2)	H12A—C12—H12B	108.0
O17—C13—C12	124.69 (18)	O14—C15—H15A	110.4
N7—C6—H6	117.1	O14—C15—H15B	110.4
N7—C6—C5	125.9 (2)	O14—C15—C16	106.64 (18)
C5—C6—H6	117.1	H15A—C15—H15B	108.6
N1—C5—C6	126.61 (19)	C16—C15—H15A	110.4
N1—C5—C4	107.45 (17)	C16—C15—H15B	110.4
C4—C5—C6	125.9 (2)	C8—C10—H10	119.9
C9—C8—N7	118.06 (19)	C9^i^—C10—C8	120.20 (18)
C9—C8—C10	119.07 (18)	C9^i^—C10—H10	119.9
C10—C8—N7	122.86 (18)	C5—C4—H4	126.0
C8—C9—H9	119.6	C5—C4—C3	108.1 (2)
C8—C9—C10^i^	120.71 (19)	C3—C4—H4	126.0
C10^i^—C9—H9	119.6	C2—C3—C4	106.86 (18)
N1—C2—H2	125.4	C2—C3—H3	126.6
N1—C2—C3	109.23 (19)	C4—C3—H3	126.6
C3—C2—H2	125.4	C15—C16—H16A	109.5
N1—C11—H11A	109.2	C15—C16—H16B	109.5
N1—C11—H11B	109.2	C15—C16—H16C	109.5
N1—C11—C12	111.88 (17)	H16A—C16—H16B	109.5
H11A—C11—H11B	107.9	H16A—C16—H16C	109.5
C12—C11—H11A	109.2	H16B—C16—H16C	109.5
			
O14—C13—C12—C11	−174.17 (16)	C5—N1—C11—C12	79.5 (2)
N1—C5—C4—C3	0.5 (2)	C5—C4—C3—C2	−0.8 (2)
N1—C2—C3—C4	0.9 (2)	C8—N7—C6—C5	179.01 (17)
N1—C11—C12—C13	169.07 (17)	C9—C8—C10—C9^i^	1.3 (3)
O17—C13—C12—C11	5.8 (3)	C2—N1—C5—C6	−177.15 (19)
N7—C6—C5—N1	−2.6 (3)	C2—N1—C5—C4	0.0 (2)
N7—C6—C5—C4	−179.3 (2)	C2—N1—C11—C12	−98.6 (2)
N7—C8—C9—C10^i^	179.47 (17)	C11—N1—C5—C6	4.5 (3)
N7—C8—C10—C9^i^	−179.52 (18)	C11—N1—C5—C4	−178.28 (18)
C13—O14—C15—C16	176.56 (17)	C11—N1—C2—C3	177.83 (18)
C6—N7—C8—C9	−134.2 (2)	C15—O14—C13—O17	−2.2 (3)
C6—N7—C8—C10	46.7 (3)	C15—O14—C13—C12	177.75 (16)
C6—C5—C4—C3	177.72 (19)	C10—C8—C9—C10^i^	−1.4 (3)
C5—N1—C2—C3	−0.6 (2)		

Symmetry code: (i) −*x*+1/2, −*y*+3/2, −*z*.
