# Supplementary figures and images for: Crystal structure of diethyl 3,3′-{2,2′-(1E)-[1,4-phenyl­enebis(azan-1-yl-1-yl­idene)]bis­(methan-1-yl-1-yl­idene)bis­(1H-pyrrole-2,1-di­yl)}di­propano­ate
Source: Acta Crystallogr E Crystallogr Commun. 2015 Mar 25;71(Pt 4):o259–60. doi: 10.1107/S2056989015005113 (PMC4438852; doi:10.1107/S2056989015005113)

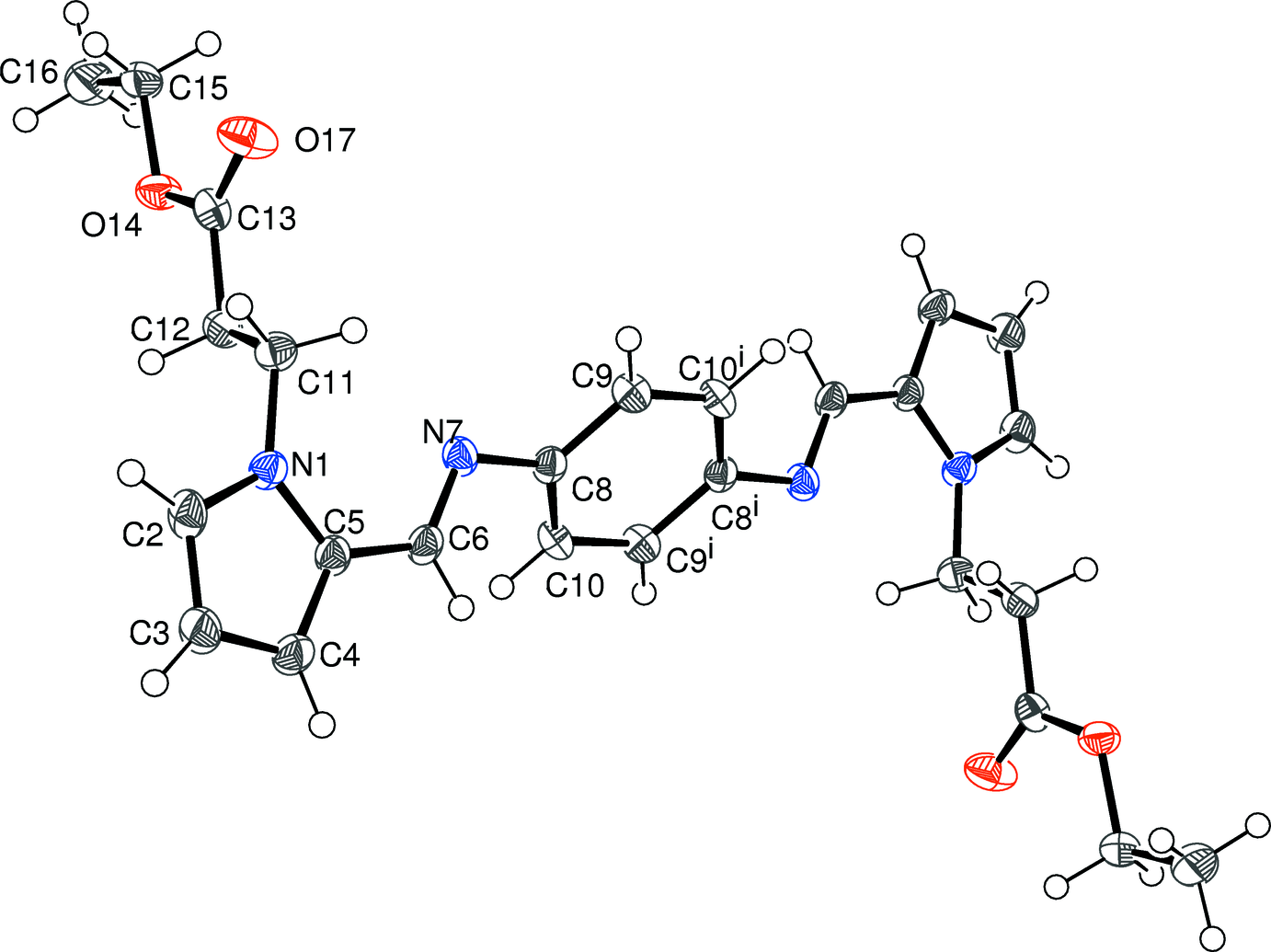

Supplement: Supplementary file 3 [file e-71-0o259-fig1.tif]
